# Exploring Genetic Diversity and Inter-/Intraspecific Polymorphism in *Rheum* sp. (Polygonaceae) Using the iPBS Retrotransposon Marker System

**DOI:** 10.3390/ijms26188943

**Published:** 2025-09-13

**Authors:** Oxana N. Khapilina, Ainur S. Turzhanova, Nadezhda G. Gemejieva, Aidar A. Sumbembayev, Raya B. Arysbayeva, Saule Magzumova, Nataliya O. Kudrina, Timur E. Kulmanov, Aigerim Mamirova, Nina V. Terletskaya

**Affiliations:** 1 National Center for Biotechnology, Korghalzhyn 13, 010000 Astana, Kazakhstan; turzhanova-ainur@mail.ru (A.S.T.); magzumovas@list.ru (S.M.); 2 Institute of Botany and Phytointroduction, Timiryazev 36, 050040 Almaty, Kazakhstan; ngemed58@mail.ru (N.G.G.); rarysbaeva@list.ru (R.B.A.); 3 Altai Botanical Garden, Ermakov 1, 071300 Ridder, Kazakhstan; aydars@list.ru; 4 Faculty of Biology and Biotechnology, Al-Farabi Kazakh National University, Al-Farabi 71/19, 050040 Almaty, Kazakhstan; kudrina_nat@mail.ru (N.O.K.); aigerim.mamirova@mail.com (A.M.); 5 Institute of Genetic and Physiology, Al-Farabi 93, 050040 Almaty, Kazakhstan; kulmanovlux@mail.ru

**Keywords:** *Rheum* species, retrotransposon marker system, polymorphism, *Rh. compactum*

## Abstract

This study investigated interspecific and intraspecific polymorphism in *Rheum* (Polygonaceae) from Kazakhstan using the inter-primer binding site (iPBS) retrotransposon marker system. The results revealed considerable variation in the level and nature of genetic polymorphism both within and among *Rheum* species and ecopopulations across different regions of Kazakhstan. *Rh. compactum* and the ecopopulation *Rh. tataricum* from the Zhambyl Region (ZH) exhibited the lowest levels of polymorphism, supporting their designation as conservation priorities. Genetic differentiation analysis among species and ecopopulations identified clear distinctions, resulting in the formation of well-defined clusters with high bootstrap support. Minimal genetic distances were observed between the two ecopopulations of *Rh. tataricum*, along with a high degree of intraspecific genetic homogeneity in *Rh. compactum* and *Rh. nanum*. A distinct genetic divergence between *Rh. compactum* and the other taxa was detected, reinforcing its status as a separate species rather than a synonym of *Rh. altaicum*. The iPBS markers proved effective for investigating genetic variation in *Rheum*, offering valuable insights for future studies aimed at understanding the evolutionary history of the genus.

## 1. Introduction

The genus *Rheum* L. (family Polygonaceae) comprises approximately 60 species of perennial herbaceous plants predominantly distributed across the temperate and subtropical regions of Asia, particularly in mountainous and arid areas. The highest species diversity is observed in China, which hosts 38 species, including 19 endemics. *Rheum* species are primarily found in Central Asia and are recognised for their significant economic and pharmacological value [1,2,3,4]. Officially recognised medicinal species such as *Rh. compactum* and *Rh. tataricum* possess haemostatic, astringent, laxative, antitumor, vitamin-rich, and anti-inflammatory properties [4]. However, several *Rheum* species are currently at risk of extinction due to unregulated harvesting and overexploitation by the pharmaceutical industry, underscoring the urgent need to investigate their genetic diversity [5]. Previous studies on *Rheum* have mainly focused on species distribution [6,7], chemical composition [8,9], and pharmacological properties [10,11,12]. However, evaluating genetic diversity is essential for the development of effective conservation strategies and the promotion of sustainable utilisation [13,14], especially since a species’ adaptive capacity to climate change and environmental stressors largely depends on its underlying genetic variability [15].

Advancements in molecular genetic techniques, from the use of polymorphic markers for individual identification to genome-wide analyses, have enabled detailed investigations into population-level genetic differentiation. These approaches not only facilitate the assessment of species diversity and population structure but also support the conservation of valuable genetic resources [16]. They are also applicable to practical tasks such as the evaluation of biological resources and the development of sustainable use strategies [17]. Genetic variation within and between populations of economically important, rare, and endangered species is critical for the long-term success of conservation programmes, as their survival depends on maintaining sufficient genetic diversity to adapt to environmental changes over time [13,18]. Therefore, assessing the level and distribution of genetic diversity is vital for the effective management and conservation of valuable, rare, and threatened species.

Nonetheless, the current literature indicates that the genetic differentiation and population structure of *Rheum* species remain insufficiently explored, limiting the implementation of effective conservation, breeding, and introduction strategies [19]. Some notable progress has been made using chloroplast and nuclear genome markers to analyse haplotype phylogenies in *Rh. pumilum* and *Rh. palmatum* [20,21]. Microsatellites, or simple sequence repeats (SSR) markers have revealed high genetic variation within and among populations of *Rh. tanguticum* [1], leading to ex situ conservation recommendations for *Rh. palmatum* and *Rh. tanguticum* in China [22]. Inter-simple sequence repeat (ISSR) markers identified low genetic diversity in populations of *Rh. emodi* in the Kashmir Himalayas, suggesting the potential loss of germplasm without timely protection [23]. Additionally, morphological and genetic diversity was evaluated in 13 geographically distant populations of *Rh. australe* in the western Himalayas [24], while SSR and ISSR markers have demonstrated genetic variation in valuable breeding traits of *Rh. ribes* [25]. Random amplified polymorphic DNA (RAPD) markers have provided insights into pedigree relationships among twelve culinary *Rheum* varieties [26]. Moreover, complete chloroplast genome analysis of *Rh. palmatum* revealed intraspecific divergence likely influenced by climatic anomalies during the Quaternary period [27]. Despite such progress, phylogenetic relationships within the genus *Rheum* remain unresolved, even with modern sequencing technologies, karyotyping, and pollen morphology analyses [27].

Among the most promising recent approaches for assessing genetic diversity is the use of retrotransposon-based molecular markers. Retrotransposable elements are ubiquitous in plant genomes and typically constitute approximately 50%, and in some cases up to 90%, of the total genome [28]. These mobile genetic elements integrate into new genomic loci, thereby generating polymorphism [29]. A significant proportion of long terminal repeat (LTR) retrotransposons is involved in various epigenetic processes, including stress response, gene regulation, DNA methylation, histone modification, and chromatin remodelling [30,31,32,33,34,35], all of which contribute to plant adaptive potential. Retrotransposons can be activated by diverse stimuli such as polyploidisation and biotic and abiotic stress, triggering transcription and mobilisation that may enhance adaptability [36]. Under environmental stress, LTR retrotransposons exhibit increased activity, resulting in mutational and insertional polymorphisms. Molecular markers derived from these elements represent a highly polymorphic and efficient system for genetic analysis. In particular, LTR retrotransposons significantly contribute to intraspecific phenotypic variability, and their transposition dynamics are closely tied to plant evolution [37].

The iPBS-PCR technique enables amplification of genomic regions flanking retrotransposons using primers complementary to conserved primer binding site (PBS) regions [38]. PBS markers offer several advantages: they do not require prior genomic information, exhibit high reproducibility and informativeness, and have been successfully applied in both inter- and intraspecific polymorphism studies across a wide range of plant taxa [39,40,41]. The primers used for iPBS amplification DNA are universal and applicable to all eukaryotic organisms [29,42]. Polymorphism is typically detected via agarose gel electrophoresis, which is simple and cost-effective. Numerous studies have confirmed that the iPBS technique provides high levels of polymorphism and reproducibility across diverse plant groups [43,44,45,46,47,48,49].

To date, several studies have successfully applied dominant iPBS markers to evaluate genetic diversity and differentiation in plants [40,50,51,52,53,54]. However, to our knowledge, this is the first study to employ the iPBS method as a molecular marker for differentiating *Rheum* species. This approach is considered highly promising for investigating inter- and intraspecific polymorphism within this relatively small genus. Therefore, the objectives of this study were: (i) to assess the level and nature of genetic polymorphism within and among *Rheum* species and ecopopulations from various regions of Kazakhstan using iPBS markers; (ii) to evaluate genetic differentiation among species and ecopopulations and analyse their relationships; and (iii) to determine whether the studied taxa conform to their current taxonomic classification as distinct species.

## 2. Results

### 2.1. iPBS Profiling of Rheum Species and Ecopopulations

The analysis of genetic diversity indices, including the average number of alleles per locus (Na), effective number of alleles (Ne), Shannon’s information index (I), expected heterozygosity (He), and unbiased expected heterozygosity (uHe), revealed substantial interspecific variability among the studied *Rheum* sp. taxa (Table 1).

The highest values across all indices were observed in *Rh. tataricum* (TUR) (Na = 1.350; Ne = 1.376; I = 0.335; He = 0.222; uHe = 0.234), indicating a relatively high level of genetic diversity at the amplification loci in this species. Similarly, elevated values were recorded for *Rh. altaicum* (Na = 1.263; Ne = 1.298; I = 0.284; He = 0.183; uHe = 0.193).

The lowest genetic diversity was observed in *Rh. compactum*, which showed the minimal values across all parameters (Na = 0.888; Ne = 1.117; I = 0.183; He = 0.115; uHe = 0.121). The highest number of unique alleles, those specific to a single species, was detected in *Rh. tataricum* (TUR), *Rh. cordatum*, *Rh. altaicum* and *Rh. nanum*, (two unique alleles), while no rare alleles were identified in *Rh. compactum* and *Rh. tataricum* (ZH).

According to the analysis of molecular variance (AMOVA), 40% of the total genetic variation was attributed to interspecific differences, whereas 60% resulted from variation within species (Table 2).

The total genetic variance was 14.709, of which 5.931 was attributed to interspecific variation and 8.778 to intraspecific variation. The differentiation coefficient (PhiPT) was 0.403 (*p* < 0.001), indicating a substantial level of genetic differentiation among the studied *Rheum* species.

### 2.2. Statistical Analysis of iPBS Profiling Data of Rheum Species and Ecopopulations

Pairwise analysis of genetic distances between populations and species of the genus *Rheum* revealed marked differences in the degree of differentiation (Table 3). The lowest genetic distance was observed between the ecopopulations *Rh. tataricum* (ZH) and *Rh. tataricum* (TUR) (0.081), indicating a high level of genetic similarity. Similarly, low values were recorded between *Rh. tataricum* (ZH) and *Rh. altaicum* (0.083), *Rh. tataricum* (ZH) and *Rh. turkestanicum* (0.090).

The most pronounced genetic differences were observed between *Rh. compactum* and the other taxa. The highest genetic distance was recorded between *Rh. compactum* and *Rh. turkestanicum* (0.911), followed by *Rh. compactum* and *Rh. tataricum* (TUR) (0.907). Similarly, high values were observed between *Rh. nanum* and other species, particularly in comparisons with *Rh. cordatum* (0.904).

**Figure 1:**
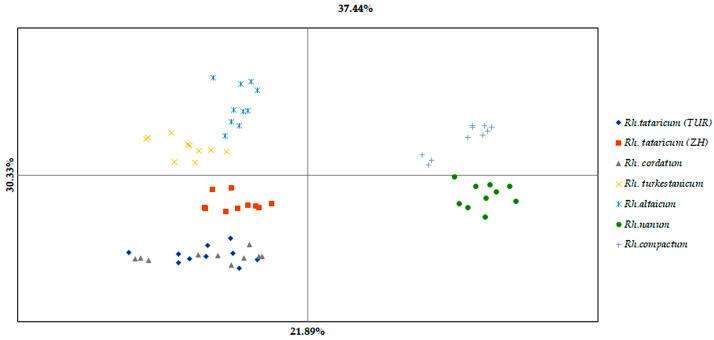
Principal coordinate analysis (PCoA) of *Rheum* species based on iPBS profiling data.

To investigate the structure of genetic diversity among *Rheum* species, principal coordinate analysis (PCoA) was conducted based on the genetic distance matrix (Figure 1). The first three axes, which represent the major directions of genetic variation among *Rheum* samples, revealed underlying patterns of diversity within species. The first principal coordinate, accounting for 21.9% of the total variation, showed that *Rh. compactum* and *Rh. nanum* are the most genetically divergent from other species. The second coordinate further differentiated *Rheum* species and highlighted genetic similarity between *Rh. tataricum* and *Rh. cordatum*, with the cumulative contribution of the first two axes reaching 30.3%.

Overall, the graphical interpretation of the PCoA results confirms a clear genetic divergence of *Rh. compactum* from the other taxa. These findings are in agreement with the AMOVA and pairwise genetic distance analyses.

**Figure 2:**
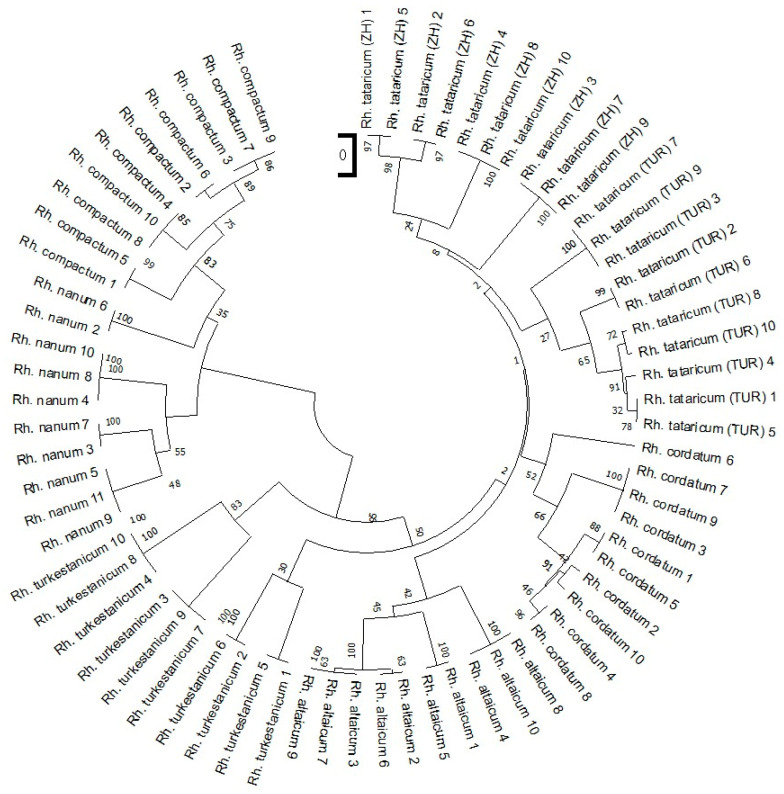
UPGMA dendrogram showing genetic relationships among *Rheum* species based on iPBS marker data. Notes: Bootstrap support values are interpreted as follows: >70%—high support; 50–70%—moderate support; <50%—low support.

To assess the genetic relationships among *Rheum* species, a dendrogram was constructed using the Unweighted Pair Group Method with Arithmetic Mean (UPGMA). The clustering was based on iPBS profiling data derived from primers targeting polymorphic retrotransposon regions (Figure 2).

The dendrogram, comprising two principal clusters, clearly reflects the grouping of samples according to species identity and corresponds well with their distribution along the PCoA axes. The upper, larger cluster is further divided into two clades: one consists of *Rh. turkestanicum* samples, which are separated from the second clade by a high bootstrap value of 99%. The second clade includes *Rh. tataricum*, *Rh. altaicum*, and *Rh. cordatum*. Within this clade, the two populations of *Rh. tataricum*, TUR and ZH, form a distinct subcluster, supported by bootstrap values ranging from 18% to 62%. The lower cluster contains *Rh. compactum* and *Rh. nanum* samples, which are grouped together with strong bootstrap support values ranging from 96% to 100%.

**Figure 3:**
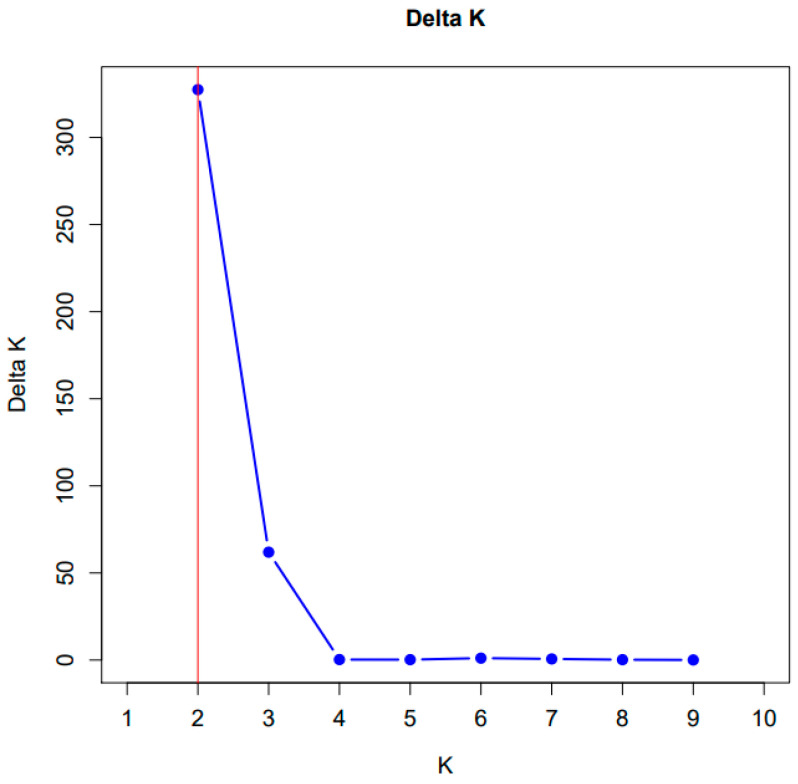
Results of Bayesian assignment analysis conducted using the STRUCTURE software. The Delta K (Δ*K*) values were plotted against various assumed numbers of clusters (*K*), identifying *K* = 2 (red line) as the most likely number of genetic clusters.

**Figure 4:**
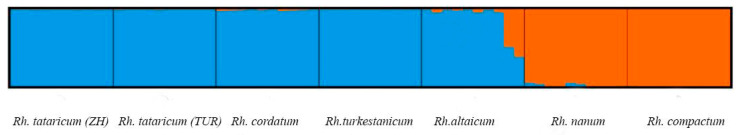
Genetic structure of five *Rheum* species and two ecopopulations of *R. tataricum*, as inferred using the STRUCTURE software based on iPBS marker data. Different colors represents the estimated proportion of membership in the two genetic clusters. Segments of each colors show extent of admixture in an individual.

In addition, population structure was assessed using the STRUCTURE software (Figure 3 and Figure 4). Based on the highest Delta K value, the optimal number of clusters was determined to be Δ*K* = 2. The peak height indicates the extent of population structure.

The impurity distribution plot generated by the STRUCTURE program for *K* = 2 represents the highest level of structure identified using the Evanno method. The results of *K* value determination by the Evanno method, along with the calculated impurity fraction (Q), are presented in Figures S6 and S7, Table S1.

## 3. Discussion

Biodiversity is increasingly threatened by factors such as salinisation, pollution, urbanisation, rising temperatures, and global climate change [55]. In this context, understanding the diversity and distribution of plant species, particularly those of economic and medicinal importance, has become critically important to for meeting the needs of a growing global population. Most studies on the impact of environmental stressors on plants have traditionally focused on phenotypic variation, physiological mechanisms of stress resistance, and the identification of stress-responsive genes. These investigations have yielded important findings, including significant correlations between morphometric traits and abiotic environmental factors [56,57,58].

In Kazakhstan, *Rheum* species are reported to have low adaptive potential and a narrow ecological range [59]. Extensive research has been conducted on their phytochemical profiles [60,61] and karyological characteristics [62]. For instance, transcriptome analysis of *Rh. australe* has revealed genes involved in signalling, transport, secondary metabolite biosynthesis, phytohormone regulation, and cellular defence, genes that likely contribute to adaptation within specific ecological niches [63]. Similarly, studies on *Rh. spiciforme* have examined responses to low-temperature stress, including reactive oxygen species (ROS) accumulation, antioxidant activity, phenolic compound synthesis, and regulatory gene expression. These findings may inform future strategies for enhancing the production of target metabolites under controlled conditions [64].

At the core of natural biodiversity, and of all plant–environment interactions, lies genetic polymorphism. Recent research increasingly emphasises the molecular and evolutionary mechanisms underlying these interactions, with a focus on the genetic diversity that supports adaptive capacity [65].

A reliable and well-optimised methodology is essential for the accurate analysis of genetic polymorphism, especially when working with herbarium material, which often contains degraded DNA and high concentrations of secondary metabolites. In this study, DNA extraction posed a significant challenge, as a large proportion of the samples consisted of herbarium-derived rhizome fragments. To overcome this, we optimised protocols for DNA extraction from dried *Rheum* material. Each protocol was evaluated based on DNA purity and PCR amplification success. The modified sodium sulphite method yielded the highest DNA purity and concentration (Figure S1). Electropherograms demonstrated that samples treated with a buffer containing 1% Na_2_SO_3_ and polyvinylpyrrolidone (PVP) exhibited distinct genomic DNA bands with good migration, indicating successful recovery. DNA recovery was 100% using both tested protocols; however, the modified method consistently produced higher purity, with A260/280 ratios ranging from 1.85 to 1.90. In contrast, the conventional cetyltrimethylammonium bromide (CTAB) method resulted in lower yields, suggesting contamination with polysaccharides and phenolic compounds, as well as possible partial degradation. A260/280 ratios in these samples ranged from 1.70 to 1.75, rendering them unsuitable for iPBS genotyping, which is highly sensitive to DNA quality. The superior performance of the sodium sulphite protocol is attributed to its reducing properties: Na_2_SO_3_ prevents the oxidation of polyphenols, which would otherwise form covalent bonds with DNA and inhibit downstream enzymatic reactions [66]. The inclusion of PVP further enhanced DNA purity by binding phenolic compounds [67].

iPBS-based markers represent an effective alternative for studying genetic polymorphism and can be applied across a wide range of plant species, including *Rheum* spp. [68]. One of their key advantages is their widespread distribution throughout the genome, which reduces the need for a large number of primers, as is often required with SSR or ISSR markers. To identify the most suitable primers for assessing genetic diversity of *Rheum*, twelve PBS primers were tested. However, only four produced spectra containing distinct polymorphic bands, each corresponding to a unique iPBS locus. The selection of these primers was based on several criteria: (i) the presence of shared bands among genetically distinct samples; (ii) the presence of polymorphic bands differentiating genetically distinct samples; (iii) the occurrence of consistent banding patterns in genetically similar samples, enabling their clustering; and (iv) the uniqueness of the iPBS profile for each species.

Amplification with the selected four primers yielded a sufficient number of polymorphic fragments to detect both interspecific and intraspecific genetic variation in *Rheum*. Furthermore, an error probability assessment using a permutation test in AMOVA confirmed the reliability of the observed genetic differentiation among species (PhiPT = 0.403, *p* < 0.001). The remaining primers produced predominantly monomorphic amplification patterns and were therefore excluded from further analysis.

The material analysed in this study was genetically homogeneous rather than highly heterogeneous. Under such conditions, where bands are either clearly present or absent, the distinction between homozygotes lacking a fragment and individuals carrying the fragment (either heterozygotes or dominant homozygotes) is readily discernible. Although it is not possible to differentiate heterozygotes from dominant homozygotes using dominant markers, the frequency of allele presence or absence can still be used to estimate population heterozygosity based on probabilistic models.

Each population examined represented a localised group of individuals assigned to a morphologically defined species. The high PhiPT value and statistically significant *p*-value confirmed that these populations were genetically distinct units, and the AMOVA results accurately reflected their underlying genetic structure. The observed genetic differentiation (*p* < 0.001) among *Rheum* populations was likely driven by genetic isolation and interspecific differences in reproductive biology.

According to evolutionary theory, plant species with restricted distribution ranges and small population sizes often exhibit low levels of genetic polymorphism and a high degree of self-compatibility. Such species typically possess fewer polymorphic loci and a lower number of alleles per locus compared to more widespread congeners [69]. Additionally, habitat fragmentation, resulting from human activities such as urbanisation, agriculture, and infrastructure development, can further reduce genetic diversity by limiting gene flow and isolating populations, ultimately compromising biodiversity and ecosystem resilience [70].

Our results demonstrated substantial interspecific variability among the studied *Rheum* taxa. In all species examined, the average number of alleles per locus (Na) exceeded the effective number of alleles (Ne), which considers allele frequency distribution. This suggests a relatively stable genetic structure, with limited but evenly distributed allelic variation within populations. However, in the context of rare and endangered species, greater emphasis should be placed on those with narrow geographic ranges. In this regard, the low heterozygosity values (He and uHe) observed in *Rh. compactum*, significantly below the dataset average, indicate reduced allelic diversity. This may reflect geographic or reproductive isolation, habitat fragmentation, restricted distribution, or the influence of genetic drift. Notably, *Rh. compactum* is confined to only three floristic regions and is listed in the Red Book of Kazakhstan as a relict species requiring strict protection [6,71].

The species *Rh. tataricum* widely distributed across Kazakhstan [6]. When comparing two ecopopulations of this species, it can be concluded that their growing conditions probably affect the level of retrotransposon polymorphism. The ecopopulation *Rh. tataricum* (TUR) demonstrated relatively higher indices of genetic diversity, indicating its high adaptability to environmental factors. However, the ecopopulation *Rh. tataricum* (ZH) demonstrated lower allelic diversity (Na and Ne), which may make it more vulnerable to environmental factors. This is probably why two ecopopulations of *Rh. tataricum* (ZH and TUR) were spatially separated in PCoA, indicating potential intraspecific divergence.

At the same time, the population of *Rh. tataricum* (TUR), as well as the species *Rh. cordatum*, *Rh. altaicum* and *Rh. nanum* (all located in the left half in PCoA) demonstrated two unique species-specific alleles, while no rare alleles were detected in *Rh. compactum* and *Rh. tataricum* (ZH). The higher number of rare alleles in some rhubarb species indicates their potential importance as a reservoir of genetic diversity for future conservation strategies.

The UPGMA dendrogram based on iPBS profiling clearly demonstrated that the studied species and ecopopulations formed well-defined, distinct clusters with strong bootstrap support, highlighting the discriminatory power of the selected markers. Although dominant markers such as iPBS may overestimate genetic differentiation, cross-validation using PCoA confirmed the robustness of the observed groupings. Pairwise genetic distance analysis among *Rheum* species and populations revealed considerable variation in the degree of genetic differentiation. The lowest genetic distance was recorded between the *Rh. tataricum* ecopopulations from Zhambyl (ZH) and Turkestan (TUR) regions (0.081), indicating a high level of genetic similarity.

Some modern databases classify *Rh. compactum* and *Rh. altaicum* as a single species, listing *Rh. altaicum* Losinsk. as the accepted name and *Rh. compactum* as a synonym [71,72,73]. However, other sources continue to recognise them as distinct species [59,74,75]. In Kazakhstan, *Rh. altaicum* is categorised as a Category III species, i.e., rare, declining in number, relict, and endemic to Eastern Kazakhstan [59]. While these two taxa are morphologically similar, the designation of *Rh. altaicum* as a synonym or subspecies of *Rh. compactum* appears poorly justified under current conditions.

Previous studies have highlighted morphological and ecological differences between these species. For instance, *Rh. altaicum* typically grows on light rocky or steppe slopes in the high-altitude Altai region, whereas *Rh. compactum* is a more forest-associated species. These differences suggest ecological divergence despite potential common evolutionary origins. It has been proposed that morphological traits in *Rheum* evolved in parallel, and that adaptation to specific environmental conditions drove the diversification of these traits [76]. In the present study, species distinctions are supported by new comparative data on genetic polymorphism. The literature indicates that genetic studies in *Rheum* species are complicated by incomplete lineage sorting (ILS) [77]. ILS results from the retention of ancestral polymorphisms, which can obscure phylogenetic relationships and may be misinterpreted as signs of recent gene flow [77,78]. While ILS is consistent with the persistence of genetic variation under selection, our findings revealed a deficit in allelic diversity for *Rh. compactum* in contrast to *Rh. altaicum*, which is confirmed by all polymorphism indices. The PCoA results further support a clear genetic divergence between *Rh. compactum* and all other taxa, including *Rh. altaicum*. These findings corroborate and complement the original morphological classification by Lozina-Lozinskaya, who described *Rheum altaicum* Losinsk. as a separate species [79].

Although dominant markers such as iPBS may overestimate genetic differentiation, cross-validation using PCoA confirmed the robustness of the observed groupings. According to the PCoA, *Rh. compactum* and *Rh. nanum* were genetically distinct from other species, with bootstrap support ranging from 76 to 96% for *Rh. compactum* and 70–100% for *Rh. nanum*. These species formed a shared lower cluster in the UPGMA dendrogram, reflecting high intraspecific genetic homogeneity. A similar pattern of isolation was observed for *Rh. turkestanicum*, which formed a separate subcluster with up to 90% bootstrap support. Thus, the UPGMA dendrogram effectively revealed both interspecific and intraspecific differentiation, which appears non-random and may reflect varying adaptive capacities among species in response to environmental conditions.

To complement and clarify the results obtained, a STRUCTURE analysis was performed. The methods used are indirectly related, as they represent different forms of sparse factor analysis [80], and together provide a more comprehensive understanding of genetic structure. Unlike other methods, the Bayesian clustering approach identifies populations by assigning individuals to one or more clusters based on the most probable admixture scenario, inferred from patterns of genetic diversity [81]. Analysis using the STRUCTURE software confirmed the division of the samples into two primary genetic clusters (∆*K* = 2), revealing the genetic closeness of *Rh. tataricum* (ZH and TUR) and the divergence of *Rh. compactum* and *Rh. nanum* from the remaining taxa. These findings are consistent with the results obtained from UPGMA and PCoA analyses. Notably, admixture was observed in 3 out of 10 *Rh. altaicum* samples (30%), and deviations from the cluster mean were found in 4 out of 10 *Rh. nanum* samples. The admixture proportions revealed in STRUCTURE for *Rh. nanum* and *Rh. altaicum*, the highest among the studied species, may indicate the degree of phylogenetic distance of these taxa. The admixture elements potentially could represent ancestral polymorphic signatures carried by mobile element sequences [81]. From a genetic perspective, it can also be hypothesised that retrotransposons in *Rheum* species respond to arid environmental conditions, which may stimulate speciation through genomic responses involving the activation of mobile elements. Such conditions likely interact with the internal regulatory mechanisms of the genome that control retrotransposon proliferation [82]. The results obtained require further clarification, including the application of additional genetic markers in future studies.

Overall, the, iPBS markers proved to be an interesting and effective tool for assessing genetic diversity in *Rheum* species.

## 4. Materials and Methods

### 4.1. Research Materials

This research was conducted as part of the interdisciplinary scientific programme “Strategy Creation for Conserving and Developing Medicinal and Veterinary Plant Resources in Kazakhstan amid Climate Change”, supported by the Committee of Science of the Ministry of Science and Higher Education of the Republic of Kazakhstan. The plant material analysed in this study comprised herbarium and dried rhizome and leaf samples of potentially medicinal species of the genus *Rheum* L. (family Polygonaceae) collected from various regions of Kazakhstan. The dataset included *Rheum turkestanicum* Janisch. (*Rh. turkestanicum*), *Rheum cordatum* Losinsk. (*Rh. cordatum*), and *Rheum tataricum* L. fil. (*Rh. tataricum*), which were collected during field expeditions carried out by staff from the Institute of Botany and Phytointroduction (Almaty, Kazakhstan). Additionally, herbarium samples of *Rheum compactum* L. (*Rh. compactum*; putative synonym: *Rh. altaicum* Losinsk.) [83] and *Rheum nanum* Lingelsh. (*Rh. nanum*), held at the National Centre for Biotechnology (Astana, Kazakhstan), were included in the analysis.

The scientific names of all plant taxa are presented in Latin and conform to the nomenclature accepted by *Plants of the World Online* [73], with synonyms provided in parentheses where relevant.

Species identification for *Rh. tataricum*, *Rh. cordatum*, and *Rh. turkestanicum* was conducted by researchers at the Institute of Botany and Phytointroduction (Almaty, Kazakhstan), while identification of *Rh. altaicum*, *Rh. nanum*, and *Rh. compactum* was carried out by specialists at the Altai Botanical Garden (Ridder, Kazakhstan) (Table 4).

The botanical descriptions of the Kazakh species of the genus *Rheum* L. presented below are based on published literature sources.

#### 4.1.1. *Rheum tataricum* L. fil.

Tatar rhubarb is a perennial herb characterised by a robust, vertical rhizome enclosed in both aged dark brown and younger dark olive sheaths. Typically, each plant bears two to three erect, hollow, glabrous, and longitudinally grooved stems. The leaves are orbicular with a cordate base and possess three prominent primary veins; the adaxial (upper) surface is glabrous, while the abaxial (lower) surface is sparsely pubescent.

The inflorescence is a globose panicle composed of flowers with five uniform, yellowish perianth lobes, each exhibiting three to five brown veins. The fruit is a narrowly obovoid nut, acutely apiculate, and ranges from dark brown to nearly black in colour. The surface is matte and finely wrinkled. The membranous wings are narrow, dark red-brown, cordate at the base, tapering apically, and bordered by a pronounced marginal vein. The perianth lobes remain appressed to the developing fruit.

**Figure 5:**
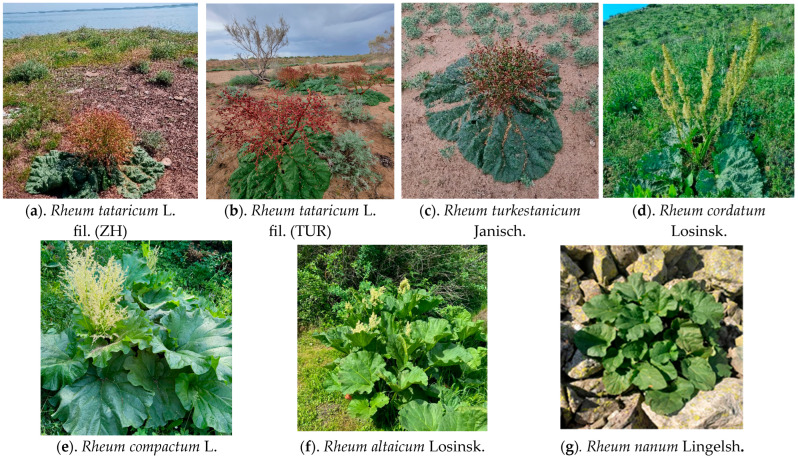
*Rheum* species included in the study. Photos by N. Gemedzhieva (**a**–**d**) and A. Sumbembaev (**e**–**g**).

This species typically occurs in desert and desert-steppe plains. Flowering takes place from April to May, with fruiting occurring from May to July [74]. The underground rhizome is the principal source of raw material for medicinal use [4]. *R. tataricum* is noted for its haemostatic, astringent, antipyretic, laxative, antitumour, and vitamin-rich properties [84,85]. It exhibits high tolerance to drought, salinity, and nutrient-poor soils. The diploid chromosome number is 2*n* = 44 [56,86,87,88,89] (Figure 5a,b).

#### 4.1.2. *Rheum turkestanicum* Janisch.

Turkestan rhubarb is a herbaceous perennial reaching 30–70 cm in height, with glabrous, longitudinally grooved stems that branch below the midpoint. The rhizome is elongated, vertically oriented, and densely covered with brown fibrous remnants of old leaf sheaths. Basal leaves are large, spreading flat along the ground, and range from broadly reniform to slightly cordate at the base, reaching up to 1.5 m in width. The upper leaf surface is glabrous, while the underside and margins are coarsely textured.

Stem leaves are reduced in size and typically number one to two per plant. The inflorescence is a broadly spreading pyramidal or nearly globose panicle. Flowers are white, and the fruits are rounded–ovoid nuts with a dark brown coloration. The fruit wings are approximately 1.5 times wider than the nut, cordate-notched at the base, and exhibit a prominent marginal vein.

Fruiting occurs from April to May. The species favours sandy habitats [74] and is predominantly found in temperate biomes. The diploid chromosome number is 2*n* = 22 [90]. The underground rhizome is used as raw material in medicinal, forage, food, and technical applications [84,85] (Figure 5c).

#### 4.1.3. *Rheum cordatum* Losinsk.

Heart-shaped rhubarb is an herbaceous perennial growing to a height of 50–100 cm. It possesses a straight, solid stem that is typically unbranched, though occasionally branched two to three times. The stem is longitudinally grooved, smooth, and reddish in colour. The rhizome is thick and horizontally oriented, bearing remnants of dark fibrous leaf sheaths.

The inflorescence is narrow and paniculate. Leaves are rounded, with a slightly pointed apex, wavy margins, and a cordate to reniform base. Each leaf features three prominently branched primary veins. The blade is glabrous, and the petioles are grooved and slightly shorter than the blade. Leaf veins are grooved, sparsely spiny in the upper portion, and smooth at the base. Basal leaves are usually one or two per plant; stem leaves are also limited to one or two and are smaller in size.

The flowers are very small, with oblong-oval, light yellow-green perianth lobes. The fruit is heart-shaped, consisting of broadly oval, heavily wrinkled dark brown nuts with reddish to light brown dry wings that taper to a relatively sharp tip.

*R. cordatum* flowers in May and grows on gravelly and rocky mountain slopes. The aerial parts of the plant are used as raw material for medicinal, nutritional, and technical purposes. Its known beneficial properties include vitamin enrichment [84,85]. The species is also used in breeding programmes aimed at developing drought- and stress-tolerant cultivars. It exhibits both diploid (2*n* = 22) and tetraploid (2*n* = 44) cytotypes [4] (Figure 5d).

#### 4.1.4. *Rheum compactum* L.

Compact rhubarb is a robust herbaceous perennial characterised by a well-developed rhizome measuring 2–5 cm in thickness. The stems are glabrous, longitudinally grooved, and can reach heights of up to 2 metres. The leaves are round-ovate, thin, and cordate at the base, with a diameter of up to 40 cm. The adaxial surface is glabrous, while the abaxial surface is sparsely pubescent, and each leaf exhibits five prominent palmate veins.

The inflorescence is dense and paniculate, with basal branches forming distinct whorls. The flowers are white and measure approximately 2.5 mm in length. The fruits are ovoid, measuring up to 11 mm long and 6–8 mm wide. The nuts are dark brown and shiny, with wings of equal width (2.5–3 mm), each bearing a central longitudinal vein.

Fruiting occurs in July. The species typically inhabits mountain slopes, riverbanks, and lakeshores [74]. *Rh. compactum* is utilised as a source of raw material for medicinal, food, and technical applications. Young stems and leaf petioles are consumed as food, while the roots may be used in leather tanning. The chromosome number is 2*n* = 44 [85] (Figure 5e).

#### 4.1.5. *Rheum altaicum* Losinsk.

Altai rhubarb is an herbaceous perennial characterised by a straight, finely grooved, hollow, leafy stem measuring 15–50 cm in height and 1–1.5 cm in thickness. The leaves are ovoid-triangular, 20–30 cm long and 15–20 cm wide, slightly tapering towards the apex and cordate at the base.

The inflorescence is dense, narrow, and upright, bearing prominent individual peduncles. The flowers are small, yellowish, and arranged in clusters of 4–7. The fruits develop on uniform peduncles that are jointed at the apex. The nut is ovoid, wrinkled, and shiny, with narrow, light brown wings that are rounded at both ends and bordered by a single marginal vein.

Flowering occurs from June to July. The species is well-adapted to high-mountain environments and typically grows on light-exposed rocky and steppe slopes rather than in forested areas [74]. The underground parts are rich in tannins and anthraquinones and are used as raw material [85].

*Rh. altaicum* has diverse applications: medicinal, culinary (young stems and leaf petioles are consumed), and technical (roots are used in leather tanning) [91,92,93,94]. The species is also widely used in breeding programmes. The chromosome number is 2*n* = 22 or 44 [85] (Figure 5f).

#### 4.1.6. *Rheum nanum* Lingelsh.

Low rhubarb is a small herbaceous perennial characterised by a vertical rhizome covered in dry, dull, dark brown sheaths. The stem is short—reaching up to 20 cm in height—coarsely grooved, and typically branches into two main stems. The leaves are usually rounded and broader than long, with three prominent primary veins. The adaxial surface is covered with wart-like protuberances, while the abaxial surface bears small stellate villi. The leaf margin is bordered by a thin white fringe composed of wart-like structures. Petioles are grooved and shorter than the leaf blade.

The inflorescence is broad and nearly pyramidal. Flowers are borne on short, thick pedicels that are jointed at the base. The outer perianth lobes are broadly ovate, yellow with brown coloration on the lower surface, and reflexed downward during fruiting. The inner lobes are yellow with red tips, lanceolate in shape, and become appressed to the developing nut. Stamens possess short filaments.

The fruits are ovoid, dull, and black-brown, with broad pink wings that are cordate at the base and notched at the apex. A marginal vein runs along the edge of the wing and connects to the nutlet by one or two cross-veins.

Flowering occurs in May. The species typically inhabits desert steppes, scree slopes of low hills, and sandy–clayey plains [74]. The entire plant is used for medicinal, nutritional, and technical purposes [85]. Roots are traditionally used for tanning leather. The chromosome number is 2*n* = 22 [62,85] (Figure 5g).

### 4.2. Research Methods

#### 4.2.1. DNA Extraction

For genotyping, 10 plant samples were collected from natural populations. To minimise the likelihood of sampling genetically identical clones resulting from vegetative reproduction, individuals were selected at a minimum distance of 10 m from one another.

DNA extraction was performed using a modified protocol based on acidic lysis with cetyltrimethylammonium bromide (CTAB) buffer and RNase A, as described in previous studies [95,96]. An acidic CTAB extraction buffer (2% CTAB, 2 M NaCl, 10 mM Na_3_EDTA, 50 mM HEPES, pH 5.3) was employed, with modifications including the use of hot chloroform to enhance the removal of proteins and polysaccharides from the plant tissue. Samples were incubated at 65 °C for 30–60 min with periodic agitation to promote complete cell lysis. Following incubation, an equal volume (700 μL) of preheated (65 °C) chloroform–isoamyl alcohol (24:1) was added to each sample. After centrifugation, the aqueous phase was carefully transferred to a new tube, and DNA was precipitated using isopropanol.

In this study, we further optimised the extraction protocol by employing a double-strength CTAB buffer supplemented with sodium sulphite (1% Na_2_SO_3_) and polyvinylpyrrolidone (PVP) to improve DNA yield and purity [97]. Dried plant material was processed using this modified acidic CTAB protocol, which included a two-fold concentrated buffer (2.0 M Tris-HCl, pH 8.0; 5.0 M NaCl; 0.5 M EDTA; 2% CTAB), along with 1% (*w*/*v*) PVP-40, and 1% (*w*/*v*) Na_2_SO_3_. Pre-homogenised samples were incubated at 65 °C for 30–60 min with occasional mixing. Subsequent purification steps followed the protocol described earlier [90]. The resulting DNA pellet was washed with 70% ethanol, air-dried at room temperature, and resuspended in 50–100 μL of TE buffer (10 mM Tris-HCl, 1 mM EDTA, pH 8.0). The efficiency of DNA extraction was assessed both qualitatively by electrophoresis on 1% agarose gel and quantitatively using spectrophotometric parameters.

#### 4.2.2. iPBS Profiling of Rheum Species

The genomic DNA profiles of *Rheum* species, extracted using the CTAB method supplemented with 1% Na_2_SO_3_ and PVP, are shown in Figure S1b. To evaluate the genetic diversity of the *Rheum* samples, a set of universal PBS primers was employed. These primers are complementary to conserved regions of diverse retrotransposons and enable the amplification of flanking genomic sequences. The sequences and characteristics of the PBS primers used in this study are presented in Table 5.

Primer efficiency was evaluated based on the uniformity of amplification profiles and the level of observed polymorphism. Following initial screening, four primers (2221, 2230, 2232, and 2240) were selected for generating rich and polymorphic amplification spectra.

Polymerase chain reactions (PCR) were performed in a total volume of 20 μL, containing 3 μL of genomic DNA (10 ng μL^−1^), 1 μL of primer (10 μM), and 1 U of Phire Plant Direct PCR Master Mix (Thermo Fisher Scientific Inc., Waltham, MA, USA). The thermal cycling conditions were as follows: initial denaturation at 98 °C for 2 min; 30 cycles of denaturation at 98 °C for 30 sec, annealing at 50–57 °C for 1 min, and extension at 72 °C for 1 min; followed by a final extension at 72 °C for 2 min. Amplification was carried out using a SimpliAmp™ Thermal Cycler (Thermo Fisher Scientific Inc., Waltham, MA, USA). To ensure reproducibility, each DNA sample was amplified in duplicate.

PCR products (amplicons) were separated on a 1.5% agarose gel stained with ethidium bromide. Fragment sizes were determined by comparison with a DNA size marker (Thermo Scientific GeneRuler DNA Ladder Mix, 100–10,000 bp) and visualised using the iBright™ CL1500 Imaging System (Invitrogen™, Thermo Fisher Scientific Inc., Waltham, MA, USA). Clear and distinct fragments were manually scored using DNA fingerprinting, with presence recorded as “1” and absence as “0”. Only clearly scorable bands were included in subsequent analysis, and each band of a unique size was considered to represent a distinct iPBS locus. The percentage of polymorphic amplicons relative to the total number of amplified fragments per primer was used to estimate the level of polymorphism.

All amplification and fingerprinting procedures were repeated twice for each DNA sample to ensure reproducibility. The selected primers demonstrated high efficiency in amplifying genomic DNA across the studied *Rheum* species, enabling the detection of clear interspecific polymorphism (Figures S2–S5). The number of polymorphic loci was sufficient to distinguish all samples, each characterised by a unique iPBS fragment profile. PCR product sizes ranged from 200 to 2800 base pairs.

#### 4.2.3. Data Processing

Gel profiles were scored using the binary fingerprinting method, with the presence of a band marked as “1” and absence as “0” in a binary matrix. This matrix was used to construct a dendrogram in MEGA-X software, employing the Unweighted Pair Group Method with Arithmetic Mean (UPGMA).

Key genetic diversity indices, including the number of alleles (Na), effective number of alleles per locus (Ne), Shannon’s information index (I), expected heterozygosity (He), unbiased expected heterozygosity (uHe), number of rare fragments (R), number and percentage of polymorphic loci (NPB), and the index of genetic differentiation (PhiPT), were calculated using GenAlex 6.5 [98].

All calculations were performed under the assumption of Hardy–Weinberg equilibrium and random mating, which are standard assumptions in the context of dominant marker analysis [99,100]. Analysis of molecular variance (AMOVA) among and within populations was also conducted in GenAlex 6.5 [101]. The dendrogram was constructed using the UPGMA clustering method [102].

Population structure analysis was assessed using STRUCTURE v2.3.4 [103], a Bayesian-based software that estimates the number of genetic clusters (*K*) and evaluates admixture levels among them. The analysis was conducted for *K* values ranging from 1 to 10, using an admixture model with correlated allele frequencies. Each run included a burn-in period of 100,000 iterations, followed by 500,000 Markov Chain Monte Carlo (MCMC) iterations [104].

STRUCTURE HARVESTER [105] was used to determine the most likely number of clusters using the Delta K method, as proposed by Evanno et al. [106]. Graphical visualisation of population structure was generated using the CLUMPAK online resource [107]. Based on the highest Delta K value, the optimal number of clusters was determined to be *K* = 2 for subsequent analysis and interpretation.

## 5. Conclusions

The level and nature of genetic polymorphism within and among *Rheum* species and ecopopulations from different regions of Kazakhstan, as analysed using iPBS markers, vary considerably. *Rh. compactum*, and the *Rh. tataricum* (ZH) ecopopulation exhibited the lowest levels of polymorphism, supporting their prioritisation for conservation.

The analysis of genetic differentiation among species and ecopopulations revealed significant differences and enabled the formation of well-defined clusters with high bootstrap support values. Minimal genetic distances were observed between the two ecopopulations of *Rh. tataricum*, while *Rh. compactum* and *Rh. nanum* demonstrated a high degree of intraspecific genetic homogeneity.

The results of this study clearly show genetic divergence between *Rh. compactum* and all other examined taxa, supporting its classification as an independent species rather than a synonym of *Rh. altaicum*. The use of iPBS markers proved effective for genetic studies of *Rheum* species. This retrotransposon-based approach not only revealed genetic variability among the studied species and ecopopulations but also provided valuable insights that may contribute to a broader understanding of the evolutionary history of the genus.

## Figures and Tables

**Table 1:** Genetic diversity indices of *Rheum* species and ecopopulations based on iPBS PCR-based genome fingerprinting.

Species	Na	Ne	I	He	uHe	R	NPB
*Rh. tataricum* (ZH)	1.063	1.293	0.267	0.176	0.185	0	51.25
*Rh. tataricum* (TUR)	1.350	1.376	0.335	0.222	0.234	2	65.00
*Rh. turkestanicum*	1.025	1.245	0.231	0.150	0.158	1	47.50
*Rh. cordatum*	1.050	1.253	0.230	0.151	0.158	2	46.25
*Rh. compactum*	0.888	1.117	0.183	0.115	0.121	0	41.25
*Rh. altaicum*	1.263	1.298	0.284	0.183	0.193	2	60.00
*Rh. nanum*	1.075	1.282	0.250	0.165	0.174	2	50.00
Average	1.102	1.275	0.254	0.166	0.175	1.3	51.61

Notes: Na—average number of alleles per locus; Ne—effective number of alleles per locus; I—Shannon’s information index; He—expected heterozygosity; uHE—unbiased expected heterozygosity; R—number of rare fragments; NPB—number (%) of polymorphic loci.

**Table 2:** Analysis of molecular variance (AMOVA) for *Rheum* species based on iPBS profiling data.

Variation	df	SS	MS	Est. Var.	%	PhiPT	*p* (Rand ≥ Data)
Interspecific	6	408.514	68.086	5.931	40%	0.403	0.001
Intraspecific	69	553.000	8.778	8.778	60%		
Total	69	961.514		14.709	100%		

Notes: df—degrees of freedom; SS—sum of squares; MS—mean square; Est. Var.—estimated variance; PhiPT—index of genetic differentiation among populations.

**Table 3:** Pairwise population matrix of Nei’s genetic identity among *Rheum* species.

*Rh. tataricum* (ZH)	*Rh. tataricum* (TUR)	*Rh.* *cordatum*	*Rh.* *turkestanicum*	*Rh.* *altaicum*	*Rh. nanum*	*Rh.* *compactum*	
-	0.922	0.771	0.914	0.920	0.832	0.893	*Rh. tataricum* (ZH)
0.081	-	0.766	0.892	0.889	0.809	0.907	*Rh. tataricum* (TUR)
0.260	0.267	-	0.714	0.830	0.904	0.763	*Rh. cordatum*
0.090	0.114	0.337	-	0.896	0.766	0.911	*Rh. turkestanicum*
0.083	0.118	0.187	0.110	-	0.836	0.903	*Rh. altaicum*
0.184	0.212	0.101	0.267	0.179	-	0.816	*Rh. nanum*
0.113	0.098	0.271	0.094	0.102	0.203	-	*Rh. compactum*

**Table 4:** Characteristics of the studied *Rheum* L. species samples used in the study.

No.	Species	GPS Coordinates	Altitude, m a.s.l.	Collection Sites of *Rheum* L. Species Specimens
1	*Rh. tataricum* L. (ZH)	N 43°25′32″ E 70°39′38″	441	Kazakhstan, Zhambyl Region, Talas District—northern shore of Lake Akkol, 8 km west of v. Akkol, within shrub–wormwood–grassland vegetation.
2	*Rh. tataricum* L. (TUR)	N 44°36′48″ E 68°44′04″	240	Kazakhstan, Turkestan Region, Sozak District—54 km north of v. Sozak, within saxaul (*Haloxylon* spp.) vegetation.
3	*Rh. turkestanicum* Janisch.	N 42°33′50″ E 67°56′42″	191	Kazakhstan, Turkestan Region, Otyrar District—19 km southwest of v. Koksarai, on sandy terrain.
4	*Rh. cordatum* Losinsk.	N 43°21′58″ E 75°02′15″	833	Kazakhstan, Zhambyl Region, Kordai District—6 km south of v. Kenen, on mountainous slopes.
5	*Rh. compactum* L. (putative synonym *Rh. altaicum* Losinsk.)	N 49°14′08″ E 89°11′23″	1186	Kazakhstan, Abay Region, Ulan District—southeastern part of the Kalbinsky Ridge, Kaindy Massif.
6	*Rh. altaicum* Losinsk. (putative synonym *Rh. compactum* L.)	N 49°29′59″ E 83°02′22″	830	Kazakhstan, Abay Region, Ulan District—Kalbinsky Ridge, Tainty Tract, southeast of v. Asybulak.
7	*Rh. nanum* Lingelsh.	N 47°34′40″ E 83°41′00″	495	Kazakhstan, Abay Region, Kurchum District—southwestern slope of the Kurchum Ridge, near v. Kalguty.

**Table 5:** Sequences and characteristics of the PBS primers used in the study.

No.	ID	Sequence	Tm (°C)	LC (%)	CG (%)
1	2217	ACTTGGATGTCGATACCA	50.0	88	44.4
2	2219	GAACTTATGCCGATACCA	55.0	88	44.4
3	2220	ACCTGGCTCATGATGCCA	55.0	80	55.6
4	2221	ACCTAGCTCACGATGCCA	58.0	88	55.6
5	2222	ACTTGGATGCCGATACCA	53.0	86	50.0
6	2224	ATCCTGGCAATGGAACCA	53.0	83	50.0
7	2226	CGGTGACCTTTGATACCA	53.0	83	50.0
8	2228	CATTGGCTCTTGATACCA	55.0	86	44.4
9	2230	TCTAGGCGTCTGATACCA	54.0	91	50.0
10	2231	ACTTGGATGCTGATACCA	50.0	83	44.4
11	2240	AACCTGGCTCAGATGCCA	58.9	88	55.6
12	2246	ACTAGGCTCTGTATACCA	50.0	88	44.4

Notes: Tm—melting temperature; GC—GC content composition; LC—linguistic sequence complexity.

## Data Availability

Data available upon request from corresponding authors.

## Supplementary Materials

The following supporting information can be downloaded at: https://www.mdpi.com/article/10.3390/ijms26188943/s1.

## Abbreviations

The following abbreviations are used in this manuscript:

AMOVA	Analysis of Molecular Variance
a.s.l.	Above sea level
iPBS	Inter-Primer Binding Site
SSR	Simple Sequence Repeats
ISSR	Inter-Simple Sequence Repeats
RAPD	Random Amplified Polymorphic DNA
DNA	Deoxyribonucleic Acid
LTR	Long Terminal Repeat
PCR	Polymerase Chain Reaction
PCoA	Principal Coordinate Analysis
UPGMA	Unweighted Pair Group Method with Arithmetic Mean
ROS	Reactive Oxygen Species
PVP	Polyvinylpyrrolidone
CTAB	Cetyltrimethylammonium Bromide
