# Supplementary material for: Exploring Genetic Diversity and Inter-/Intraspecific Polymorphism in Rheum sp. (Polygonaceae) Using the iPBS Retrotransposon Marker System
Source: Int J Mol Sci. 2025 Sep 13;26(18):8943. doi: 10.3390/ijms26188943 (PMC12470156; doi:10.3390/ijms26188943)
Supplement: Supplementary file 1 [file ijms-26-08943-s001.zip › ijms-3734897-supplementary.pdf]

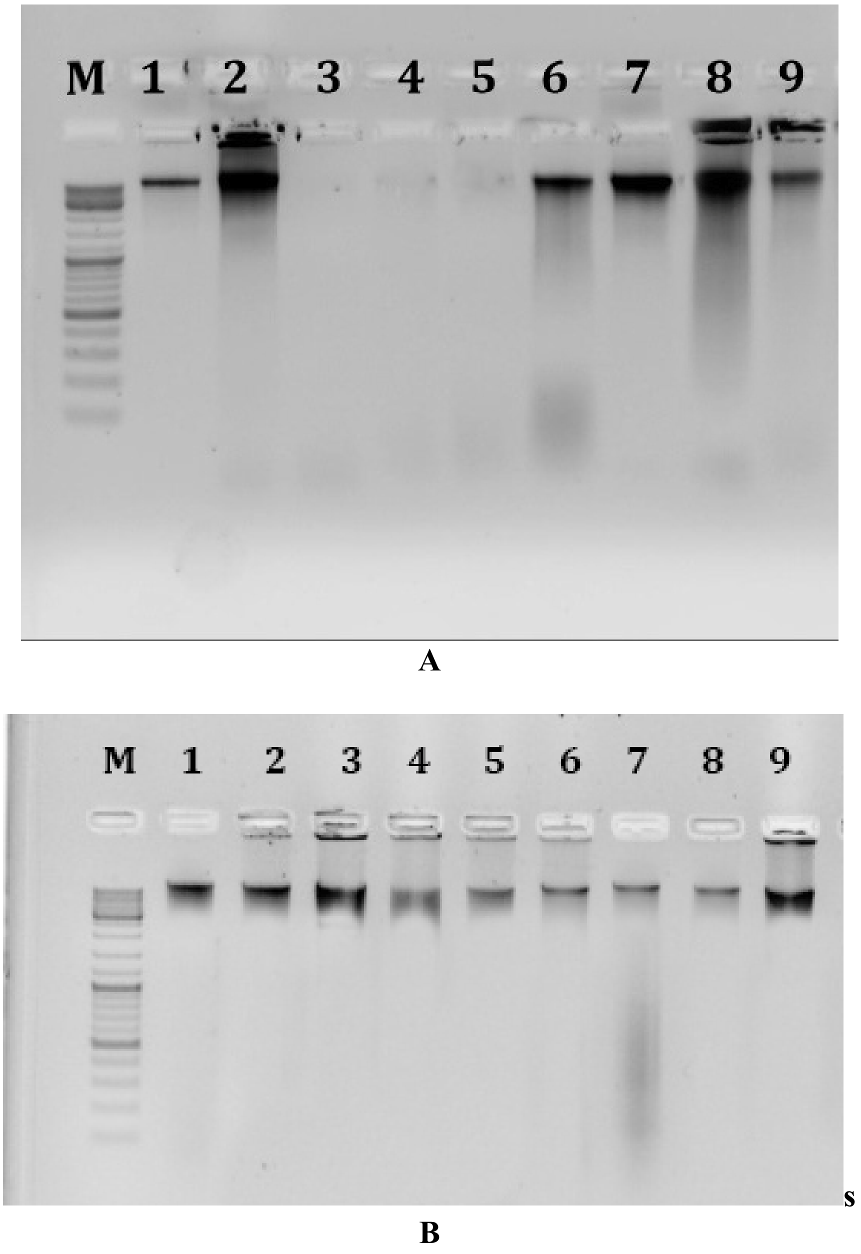

Figure S1. Genomic profile of *Rheum* sp. extracted using CTAB method (A) and with addition of Na2SO3 (1%) and PVP (B)

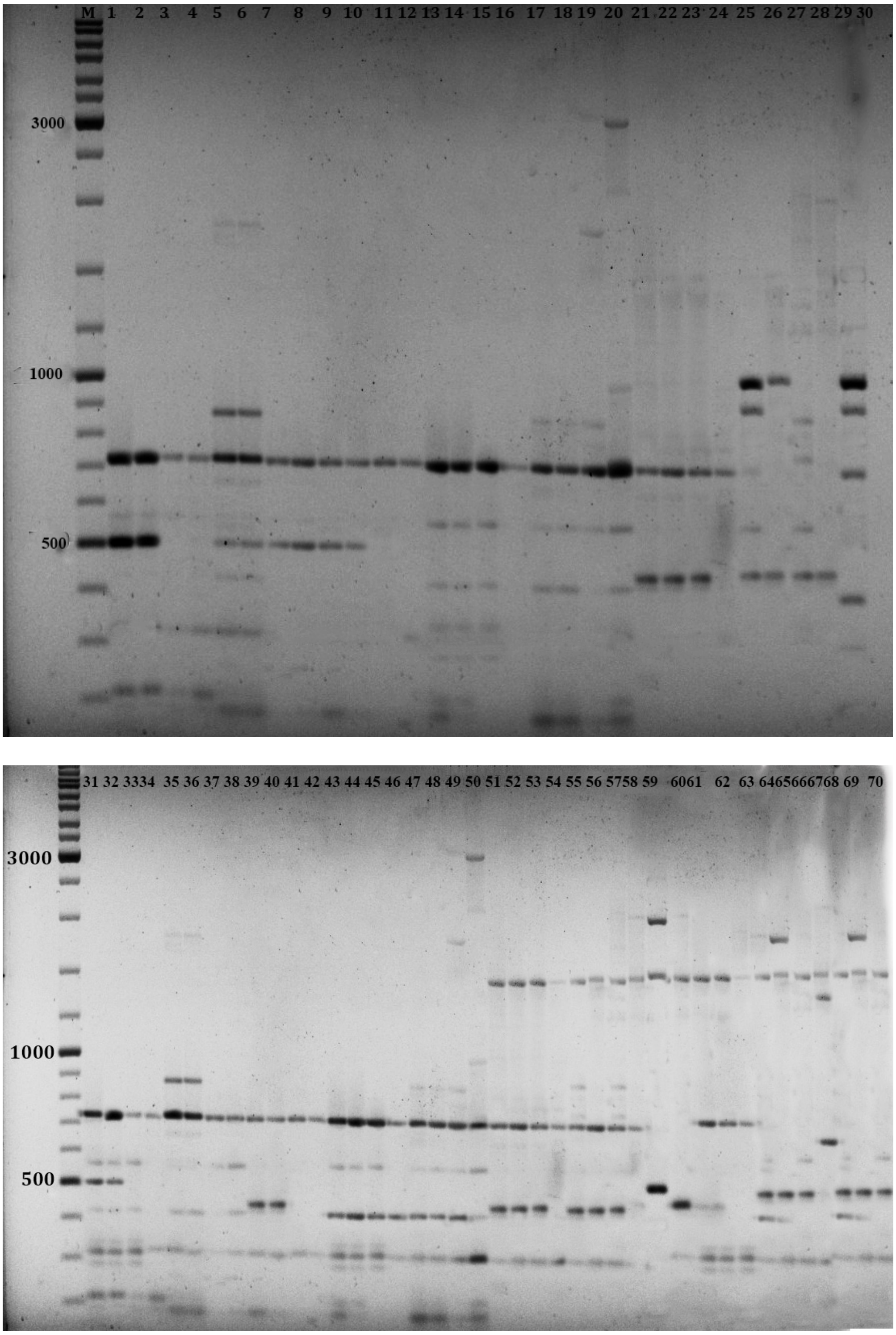

Figure S2. Electropherogram of the results of amplification of individual DNA samples from 70 samples of 7 populations of *Rheum* sp. with primer 2221

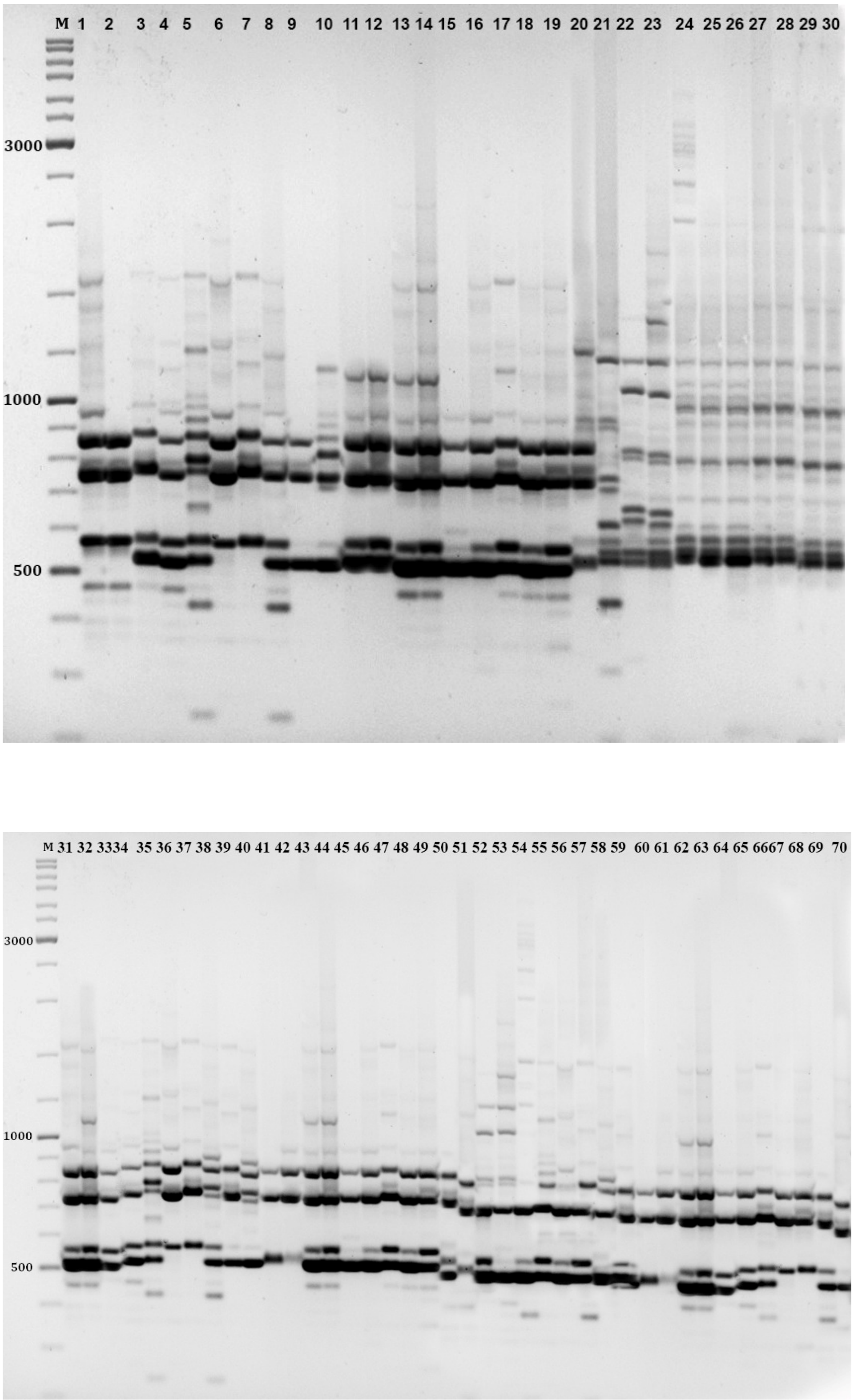

Figure S3. Electropherogram of the results of amplification of individual DNA samples from 70 samples of 7 populations of *Rheum* sp. with primer 2230

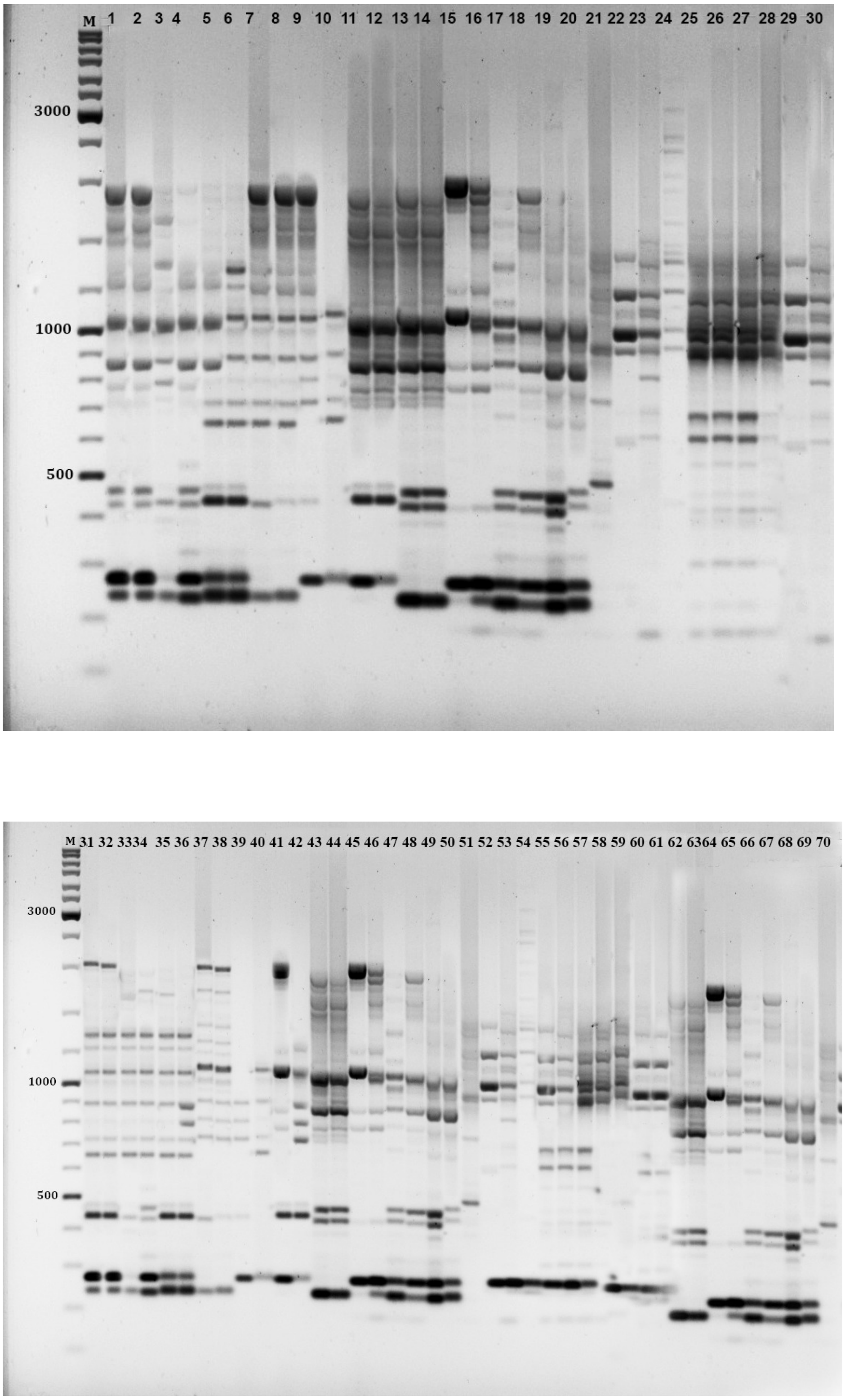

Figure S4. Electropherogram of the results of amplification of individual DNA samples from 70 samples of 7 populations of *Rheum* sp. with primer 2232

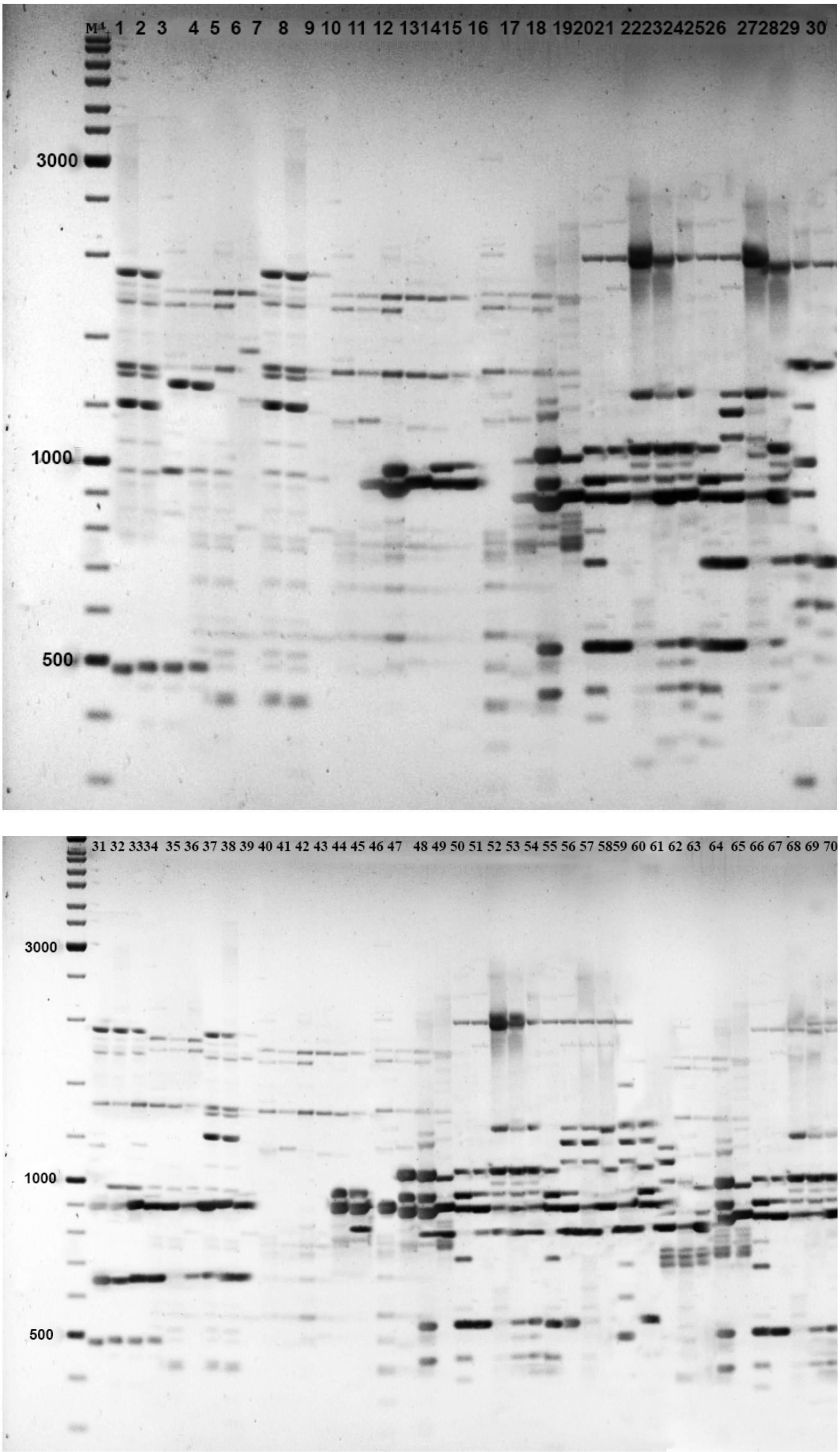

Figure S5. Electropherogram of the results of amplification of individual DNA samples from 70 samples of 7 populations of *Rheum* sp. with primer 2240

```
# File generated at 2025-07-18 11:47:31 CST
# Generated by StructureSelector
# http://lmme.qdio.ac.cn/StructureSelector/
# https://lmme.ac.cn/StructureSelector/
# Contact: Yulong Li <liyulong12@mails.ucas.ac.cn>
# Citation: Li YL, Liu JX (2018) StructureSelector: A web based software
to select and visualize the optimal number of
# clusters using multiple methods.Molecular Ecology Resources, 18:176-
177.
#
# Evanno method:
# Citation:
# Evanno G, Regnaut S, Goudet J (2005) Detecting the number of clusters
of individuals using the software structure:
# a simulation study. Molecular Ecology, 14: 2611-2620.
# K    Reps    Mean LnP(K)    Stdev LnP(K)    Ln'(K)    |Ln''(K)|
Delta K
1     10    -2671.59000 0.50211     NA    NA    NA
2     10    -1941.26000 0.34383     730.33000   572.25000   1664.31774
3     10    -1783.18000 9.78841     158.08000   3.70000     0.37800
4     10    -1628.80000 31.00642    154.38000   1.86000     0.05999
5     10    -1472.56000 18.08795    156.24000   140.05000   7.74273
6     10    -1456.37000 290.44048   16.19000    142.44000   0.49043
7     10    -1297.74000 91.44260    158.63000   127.42000   1.39344
8     10    -1266.53000 226.74166   31.21000    74.76000    0.32971
9     10    -1160.56000 222.33306   105.97000   23.50000    0.10570
10    10    -1031.09000 32.66627    129.47000   NA    NA
```

Figure S6. The results of K value determination by the Evanno method

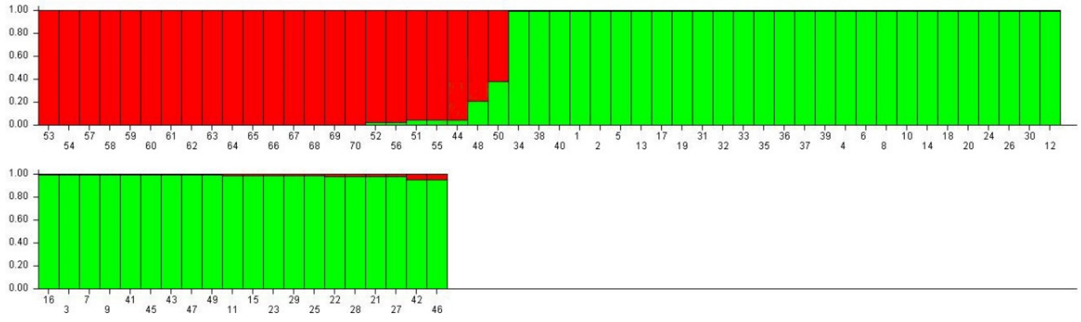

Figure S7. The calculated by STRUCTURE impurity fraction (Q)

Table S1. Estimates of admixture proportions of 7 populations of Rheum sp. based on Q values (K = 2)

Populations, species	Interferred ancestry of individuals				
***Rh. tataricum* (ZH)**		Label	(%Miss)	:	Inferred clusters
	1	1	(0)	:	0.002 0.998
	2	2	(0)	:	0.002 0.998
	3	3	(0)	:	0.006 0.994
	4	4	(0)	:	0.003 0.997
	5	5	(0)	:	0.002 0.998
	6	6	(0)	:	0.003 0.997
	7	7	(0)	:	0.006 0.994
	8	8	(0)	:	0.003 0.997
	9	9	(0)	:	0.006 0.994
	10	10	(0)	:	0.003 0.997
***Rh. tataricum* (TUR)**		Label	(%Miss)	:	Inferred clusters
	11	11	(0)	:	0.008 0.992
	12	12	(0)	:	0.004 0.996
	13	13	(0)	:	0.002 0.998
	14	14	(0)	:	0.003 0.997
	15	15	(0)	:	0.008 0.992
	16	16	(0)	:	0.004 0.996
	17	17	(0)	:	0.002 0.998
	18	18	(0)	:	0.003 0.997
	19	19	(0)	:	0.002 0.998
	20	20	(0)	:	0.003 0.997
Rh. cordatum		Label	(%Miss)	:	Inferred clusters
	21	21	(0)	:	0.020 0.980
	22	22	(0)	:	0.015 0.985
	23	23	(0)	:	0.010 0.990
	24	24	(0)	:	0.003 0.997
	25	25	(0)	:	0.011 0.989
	26	26	(0)	:	0.003 0.997
	27	27	(0)	:	0.020 0.980
	28	28	(0)	:	0.015 0.985
	29	29	(0)	:	0.010 0.990
	30	30	(0)	:	0.003 0.997
Rh. turkestanicum		Label	(%Miss)	:	Inferred clusters
	31	31	(0)	:	0.002 0.998
	32	32	(0)	:	0.002 0.998
	33	33	(0)	:	0.002 0.998
	34	34	(0)	:	0.001 0.999
	35	35	(0)	:	0.002 0.998
	36	36	(0)	:	0.002 0.998
	37	37	(0)	:	0.002 0.998
	38	38	(0)	:	0.001 0.999
	39	39	(0)	:	0.002 0.998
	40	40	(0)	:	0.001 0.999

Rh. altaicum	Label (%Miss) : Inferred clusters<br>41 41 (0) : 0.006 0.994<br>42 42 (0) : 0.043 0.957<br>43 43 (0) : 0.007 0.993<br>44 44 (0) : 0.969 0.031<br>45 45 (0) : 0.006 0.994<br>46 46 (0) : 0.043 0.957<br>47 47 (0) : 0.007 0.993<br>48 48 (0) : 0.819 0.181<br>49 49 (0) : 0.007 0.993<br>50 50 (0) : 0.619 0.381
Rh. nanum	Label (%Miss) : Inferred clusters<br>51 51 (0) : 0.967 0.033<br>52 52 (0) : 0.973 0.027<br>53 53 (0) : 0.998 0.002<br>54 54 (0) : 0.998 0.002<br>55 55 (0) : 0.967 0.033<br>56 56 (0) : 0.972 0.028<br>57 57 (0) : 0.998 0.002<br>58 58 (0) : 0.998 0.002<br>59 59 (0) : 0.998 0.002<br>60 60 (0) : 0.998 0.002
Rh. compactum	Label (%Miss) : Inferred clusters<br>61 61 (0) : 0.998 0.002<br>62 62 (0) : 0.998 0.002<br>63 63 (0) : 0.998 0.002<br>64 64 (0) : 0.998 0.002<br>65 65 (0) : 0.998 0.002<br>66 66 (0) : 0.998 0.002<br>67 67 (0) : 0.998 0.002<br>68 68 (0) : 0.998 0.002<br>69 69 (0) : 0.998 0.002<br>70 70 (0) : 0.998 0.002
